# In Vitro Biological Impact of Nanocellulose Fibers on Human Gut Bacteria and Gastrointestinal Cells

**DOI:** 10.3390/nano10061159

**Published:** 2020-06-12

**Authors:** Viviana R. Lopes, Maria Strømme, Natalia Ferraz

**Affiliations:** Nanotechnology and Functional Materials, Department of Materials Science and Engineering, Uppsala University, Box 534, 751 21 Uppsala, Sweden; vivianarlopes@gmail.com (V.R.L.); maria.stromme@angstrom.uu.se (M.S.)

**Keywords:** nanofibrillated cellulose, oral toxicity, nanotoxicity, surface chemistry, normal intestinal flora

## Abstract

Wood-derived nanofibrillated cellulose (NFC) has long been recognized as a valuable nanomaterial for food-related applications. However, the safety of NFC cannot be predicted just from the chemical nature of cellulose, and there is a need to establish the effect of the nanofibers on the gastrointestinal tract, to reassure the safe use of NFC in food-related products. The present work selected the intestinal cells Caco-2 and the gut bacteria *Escherichia coli* and *Lactobacillus* *reuteri* to evaluate the in vitro biological response to NFC. NFC materials with different surface modifications (carboxymethylation, hydroxypropyltrimethylammonium substitution, phosphorylation and sulfoethylation) and unmodified NFC were investigated. The materials were characterized in terms of surface functional group content, fiber morphology, zeta potential and degree of crystallinity. The Caco-2 cell response to the materials was evaluated by assessing metabolic activity and cell membrane integrity. The effects of the NFC materials on the model bacteria were evaluated by measuring bacterial growth (optical density at 600 nm) and by determining colony forming units counts after NFC exposure. Results showed no sign of cytotoxicity in Caco-2 cells exposed to the NFC materials, and NFC surface functionalization did not impact the cell response. Interestingly, a bacteriostatic effect on *E. coli* was observed while the materials did not affect the growth of *L. reuteri*. The present findings are foreseen to contribute to increase the knowledge about the potential oral toxicity of NFC and, in turn, add to the development of safe NFC-based food products.

## 1. Introduction

In the era of nanotechnology, nanocellulose (NC) has emerged as a highly interesting material for a wide range of industrial (e.g., packaging and electronics) and biomedical (e.g., wound care and tissue engineering) applications [1,2]. Moreover, the increasing concern for sustainable and environmentally friendly resources has also contributed to the growing interest of NC-based products [3]. NC consists of cellulose fibrils (nanofibrillated cellulose) or crystallites (cellulose nanocrystals) with at least one dimension in the nanoscale and combines cellulose properties, such as hydrophilicity, chirality and broad chemical-modifying capacity with specific nanomaterial characteristics like high specific surface area and high aspect ratio, together with tailorable mechanical, rheological and optical properties [4,5]. NC can be derived from a diversity of sources, including wood, algae, bacteria and tunicates. The production methods include top-down approaches, as in the case of wood-derived NC, which is obtained by chemical and/or mechanical treatments of cellulose, or bottom-up approaches, as in the case of bacterial NC, which is synthesized by certain type of bacteria from glucose [6].

Wood is the most important source of nanofibrillated cellulose (NFC). NFC individual fibrils are typically 2–10 nm in diameter and several micrometers in length, forming 20–60 nm thick aggregates [7]. The physicochemical properties of NFC materials, such as fiber dimensions, degree of crystallinity, aspect ratio, specific surface area and degree of branching of the nanofibrils, depend on the raw material, the pretreatments used in the manufacturing process and the post-manufacturing chemical modifications [7]. These materials characteristics may affect the interactions between the cellulose nanofibers and biological systems and thus the materials’ hazard to human health and the environment. 

As for other nanomaterials, it is believed that, in order to reach the full potential of NFC applications, the materials’ safety aspects have to be considered at early stages of product development [8]. In this sense, the number of studies aiming to understand the potential hazard of NC materials, including NFC, has increased during the last years [9,10]. However, as summarized by Endes et al., while several authors suggest limited toxicity, others alert about the potential hazard of NC materials [9]. Conflicting results can be a consequence of the different physicochemical properties of the NC materials under study and the fact that the route of exposure may also influence the biological impact of NC [8]. It should be noticed that the main focus has been occupational exposure, and therefore most of the toxicological studies are based on exposure by inhalation. However, the importance of assessing oral exposure to NC has been recognized, together with the need for increasing the knowledge about NC toxicity when the exposure occurs via the oral route [10]. Oral exposure is especially relevant when considering the proposed use of NFC in food additives (e.g., as calorie reducer, thickener and stabilizer) [11] and food packaging [12] (in the case that NFC in packaging migrates to food). Even though conventional forms of cellulose (e.g., carboxymethyl cellulose, CMC and E466) have been widely used in the food industry, the safety of NFC cannot be predicted just from the chemical nature of cellulose. There is a need to establish the biological impact of the nanofibers on the gastrointestinal tract, in order to reassure the safe use of NFC in food-related products [10].

The gastrointestinal tract (GIT) is a selective mucosal barrier with different functions (digestion, adsorption, secretion and protection against exogenous agents) and where symbiotic interactions between the host cells and the resident microbiota, also called intestinal normal flora, take place [13]. The intestinal normal flora aids in the digestion, enhances metabolism, promotes barrier integrity and prevents pathogens from entering mucosal tissues. Changes in the gut microbiota have been related with the development of gastrointestinal diseases, such as inflammatory bowel diseases, obesity and diseases of the respiratory tract (e.g., allergy and asthma) [13,14]. Although there are still few studies on the interactions of nanoscale materials with the gut microbiota, authors have highlighted that nanomaterials can alter the microbiota balance and the risk for clinicals disorders such as colitis and immunological disfunctions associated with the ingestion of nanomaterials [15,16]. 

In the present work, we investigate the in vitro biological impact of NFC on selected bacteria of the gut microbiota (*Escherichia coli* and *Lactobacillus reuteri*) and on intestinal cells (Caco-2 cells). In addition, we evaluate if NFC materials with different surface modifications could induce different biological responses in vitro. Surface chemical modification of the cellulose fibers is a well-established method to facilitate the nanofibrillation during production, leading to functionalized NFC with distinct properties and qualities. Moreover, chemical modifications are also exploited to endow NFC with specific properties or functions and broaden its range of applications [17]. Thus, we selected four NFC materials with the following surface functionalizations: carboxymethylated, hydroxypropyltrimethylammonium, phosphorylated and sulfoethylated NFCs, together with the unmodified NFC, to investigate the effect of nanofiber surface modification on the GIT. 

## 2. Materials and Methods 

### 2.1. NFC Materials

NFC materials, provided by RISE Bioeconomy (Stockholm, Sweden), were produced from commercial never-dried bleached sulfite softwood dissolving pulp (lignin content < 1.5%, xylose < 1.7%, mannose < 1.8%, Domsjö Fabriker AB, Stockholm, Sweden) and were biocide-free. Unmodified NFC (U-NFC) was prepared by enzymatic pretreatment of the wood pulp as previously described by Pääkoo et al. [18], while carboxymethylated NFC (referred to as anionic NFC, A-NFC) and hydroxypropyltrimethylammonium NFC (referred to as cationic NFC, C-NFC) were prepared as described by Hua et al. [19]. Phosphorylated NFC (herein denoted P-NFC) was synthesized according to the method presented by Naderi et al. [20], employing a 4:1 phosphorous:glucopyranose molar ratio and 5 microfluidizer passes at 1700 bar. Finally, sulfoethylated NFC (S-NFC) was prepared by following the protocol described by Naderi et al. [21].

**Figure 1:**
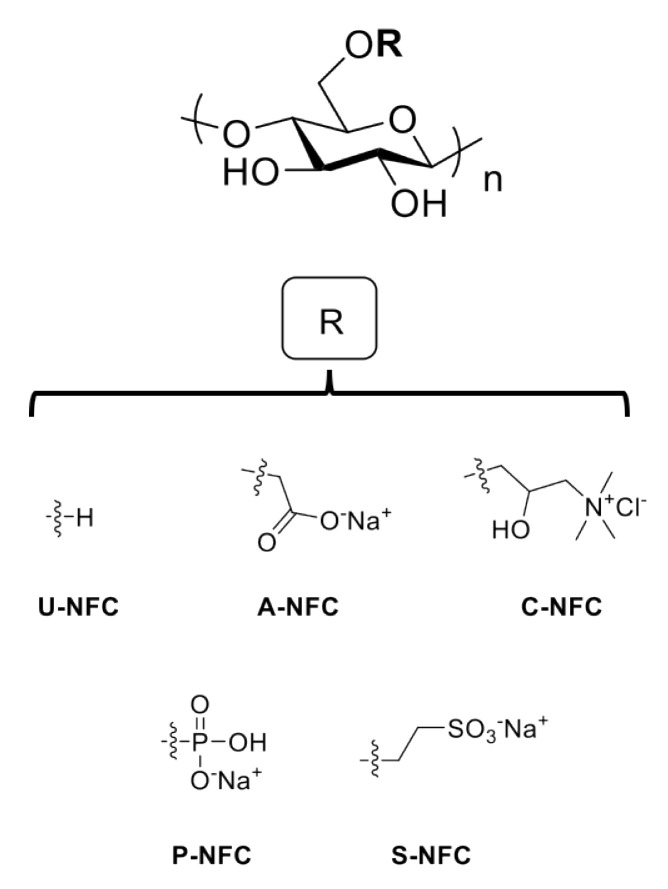
Chemical structures of the nanofibrillated cellulose (NFC) materials under study.

Figure 1 shows the chemical structures of the NFC materials under study.

### 2.2. NFC Exposure Suspensions

Suspensions of the NFC materials (U-, A-, C-, P- and S-NFC) were prepared in phosphate saline buffer (PBS) at a concentration of 5 mg/mL and dispersed through ultrasonication (70% amplitude, 12 min, Vibracell sonicator 600 W, 20 Khz, Sonics, Newtown, CA, USA) and thereafter sterilized by autoclaving (20 min, 121 °C, 15 KPa). 

The stock suspensions were further used to prepare the exposure suspensions for the cell and bacteria studies. For the cell studies, each stock suspension was diluted in cell culture medium, at the following concentrations: 500, 250, 100 and 50 μg/mL. Meanwhile, for the bacteria studies, the exposure suspensions were made in Luria-Bertani (LB) broth (for *E. coli* exposure) or Man Rogosa Sharpe (MRS) broth (for *L. reuteri* exposure) at 50 and 500 μg/mL.

### 2.3. Material Characterization

#### 2.3.1. Surface Functional Group Density

Conductimetric titration was employed to quantify the number of carboxyl groups in U-NFC and A-NFC by following the method described by Hua et al. [22]. The number of hydroxypropyltrimethylammonium (HPTMA) groups in C-NFC was determined by elemental analysis of total nitrogen, using an Antek MultiTek nitrogen, sulfur and halides Analyzer (ANTEK instruments Inc., Houston, TX, USA) and the pyro-chemiluminescence method, with urea, for calibration. The amounts of sulfate groups in S-NFC and phosphate groups in P-NFC were determined by Inductively Coupled Plasma–Optical Emission Spectroscopy (ICP–OES, Perkin Elmer Optima 8300, Perkin Elmer Inc, Waltham, MA, USA). Prior to analysis, both samples were oxidized with hydrogen peroxide and thereafter wet-digested in a microwave oven with nitric acid. 

Surface functional group density was expressed as mmol surface group per g of dry NFC for all materials under study. 

#### 2.3.2. Zeta Potential

Suspensions of 0.001% (*w*/*w*) NFC samples in 10 mM NaCl were prepared and homogenously dispersed by ultrasonication (30 s, 70% amplitude, Vibracell sonicator 600W, 20 Khz, Sonics, Newtown, CA, USA). NFC suspensions were also prepared in complete cell culture medium and in the bacteria growth broths LB and MRS at 0.001% (*w*/*w*). The electrophoretic mobility of the samples was measured at 25 °C (NaCl suspensions) or at 37 °C (cell culture medium and broth suspensions), using disposable folded capillary cells (Malvern Instruments, Malvern, UK) in a ZetaSizer Nano instrument (Malvern Instruments, Malvern, UK). The z-potentials were determined from the electrophoretic mobilities by using the Smoluchowski model [23]. 

#### 2.3.3. Transmission Electron Microscopy

The NFC materials were visualized by transmission electron microscopy (TEM). Samples were prepared by following the protocol by Usov et al. [24]. Briefly, 5 μL of NFC suspension (0.1% in deionized water) was deposited onto copper TEM grids with formvar carbon support film for 1 min, followed by the addition of 5 μL of 2% uranyl acetate for 1 s and again 5 μL of 5% uranyl acetate for 15 s. After each step, the excess of moisture was drained along the periphery, using filter paper. The grids were examined by using a FEI Titan Themis TEM operated at 200 kV. 

#### 2.3.4. Degree of Crystallinity

Powder X-ray diffraction (XRD) patterns were acquired by using a Bruker D8 Advance Eco instrument (Bruker, Bremen, Germany) with Cu-Kα radiation (λ = 1.5418 Å), with a step size of 0.016° and 1 s per step in the 2ϴ range of 5–60°. The crystallinity index (*CrI*) of the sample was calculated using the Segal peak height method [25];

$$CrI=\frac{I_{200}-I_{min}}{I_{200}}.$$

where *I*_200_ and *I_min_* are the overall intensities at 22.5° and 18.7° 2ϴ, respectively.

### 2.4. Caco-2 Study

Human epithelial colorectal adenocarcinoma cells Caco-2 (ATCC number HTB-37™, American Type Culture Collection) were cultured in Minimum Essential Medium (MEM) (Thermo Fisher Scientific, Waltham, MA, USA) supplemented with 20% (*v*/*v*) fetal bovine serum, 1% (*v*/*v*) 100 mM sodium pyruvate, 1% (*v*/*v*) non-essential amino acids, 100 IU/mL penicillin and 100 μg/mL streptomycin (all supplements from Thermo Fisher Scientific, Waltham, MA, USA). The cell cultures were kept in a 37 °C and 5% CO_2_ incubator with a humidified atmosphere.

#### Cytotoxicity Assessment

Caco-2 cells were seeded in 96-well plates (clear, flat bottom tissue culture plates, VWR International, PA, USA) at a density of 10,000 cells per well and cultured for 24 h in a 37 °C and 5% CO_2_ incubator with humidified atmosphere. Thereafter, near confluent monolayers were exposed to the NFC suspensions (U-NFC, A-NFC, C-NFC, P-NFC and S-NFC) at a concentration range of 50 to 500 μg/mL (200 μL per well) for 24 and 48 h. Non-treated cells served as the negative control, and cells exposed to 5% dimethylsulfoxide (DMSO) in cell culture medium served as the positive control.

1. Resazurin Assay

The metabolic activity of the cells was assessed by the resazurin assay after 24 and 48 h of exposure to the NFC suspensions. Cell culture medium was removed, and the cell layers were carefully washed with warm PBS before adding 200 μL of resazurin reagent (Thermofisher Scientific, Waltham, MA, USA) diluted 1:10 in cell culture medium. After 90 min incubation at 37 °C, 100 μL of each well was transferred to a black 96-well plate, and the fluorescence was measured at 560 nm excitation and 590 nm emission wavelengths, using a plate reader (Tecan Infinite M200 spectrofluorometer, Zürich, Switzerland). Cell metabolic activity was expressed as the percentage of the negative control. The experiments were conducted at least three times in triplicated wells for each NFC sample and dose. Control experiments showed that neither of the NFC samples nor the cell culture medium interfere with the assay.

2. Live/Dead Staining

A live/dead staining kit (Sigma Aldrich, USA) was used to image Caco-2 cells after NFC exposure and investigate cell integrity damage. Caco-2 cells were exposed to the highest and lowest NFC concentrations (i.e., 50 and 500 μg/mL) during 24 and 48 h. Thereafter, the cell culture medium was removed and 100 μL of PBS containing 0.2% (*w*/*w*) of calcein-AM and 0.1% (*w*/*w*) of propidium iodine was added per well and incubated for 15 min at 37 °C and 5% CO_2_. Subsequently, cells were imaged by using a fluorescence microscope (Nikon ECLIPSE TE2000-U, Tokyo, Japan) with λ_ex_ 490 nm λ_em_ 515 nm filter set for viable cells and λ_ex_ 535 nm λ_em_ 617 nm for dead cells.

### 2.5. Bacteria Study

*E. coli* ((Migula) Castellani and Chalmers, ATCC 13706) and *L. reuteri* DSM 17938 (kindly provided by BioGaia AB, Stockholm, Sweden) were used as gut bacteria model strains. The pre-culture of each bacterium was done from frozen suspensions. Each frozen stock was revived by streaking the culture onto its respective agar plate (LB agar for *E. coli* and MRS agar for *L. reuteri*) and incubated at 37 °C overnight. Afterward, an isolated single colony of each bacterium was cultured on the respective liquid broth medium under dynamic conditions (orbital shaker at ca. 200 rpm) for *E. coli* and under static conditions for *L. reuteri* (micro-aerobic conditions). 

For all studies, overnight cultures of *E. coli* and *L. reuteri* grown in LB broth and MRS broth, respectively, were used. Before the exposure experiments, bacteria concentration of the overnight cultures was determined by measuring the optical density at 600 nm (OD600), using a spectrophotometer (UV-1800, Shimadzu, Kyoto, Japan). 

#### 2.5.1. Measurement of Bacterial Growth in the Presence of NFC

Aliquots of approximately 20 μL of bacteria overnight cultures were added to the wells of 96-well plates (clear, flat bottom tissue culture plates, VWR International, PA, USA), followed by the addition of the corresponding broth to give a final volume of 200 μL per well and an initial OD600 reading of 0.1. The broths were supplemented with the NFC materials (50 μg/mL and 500 μg/mL), or added alone, in the case of the control. 

The cultures were incubated in humidified atmosphere, at 37 °C, under dynamic conditions for *E. coli* (orbital shaker at ca. 200 rpm) and under static conditions for *L. reuteri*. The bacterial growth was monitored by measuring OD600 every 30 min, during 8 h, using a plate reader (Tecan Infinite M200 spectrofluorometer, Zürich, Switzerland). Each sample was run in triplicates, and each experiment was repeated at least two times. 

Growth curves were plotted, and the number of bacterial cells at selected time points were expressed as a percentage of the control (non-exposed bacteria). The selected time points correspond to the exponential phase (3 h for *E. coli* and 4 h for *L. reuteri*) and to the last studied time point (8 h for both bacteria). 

#### 2.5.2. Colony Forming Units (CFUs) Assay

For this assay, the bacteria were exposed to the NFC materials, as described above; culture media aliquots were taken at the exponential phase (at 4 h for *L. reuteri* and 3 h for *E. coli*), serial dilutions in the corresponding broth were subsequently prepared and 10 μL of each serially diluted sample were spread on agar plates. LB agar plates were used for *E. coli* and cultured overnight at 37 °C, while MRS agar plates were used for *L. reuteri*, and the culture was done under anaerobic conditions, at 37 °C for 48 h. Anaerobic conditions were maintained by using AnaeroJar jars (Fisher, Hampton, NH, USA). The oxygen level (<1%) was monitored by using anaerobic indicator strips (Fisher, Hampton, NH, USA).

After the incubation time, CFUs were counted. Three replicates were performed for CFU assays, and each experiment was repeated three times. Data were presented as log CFU/mL, and comparisons between NFC-treated and non-treated bacteria were made to assess the effect of the NFC materials on the number of viable bacteria. 

### 2.6. Statistical Analysis

Data were analyzed by one-way analysis of variance (ANOVA), followed by Dunnett´s multiple comparison post hoc test, using GraphPad Prism 8, version 8.2.1 (GraphPad Software, San Diego, CA, USA). The *p*-values lower than 0.05 were considered statistically significant. Results are presented as the mean ± standard error of the mean (SEM).

## 3. Results and Discussion

Surface chemistry of NFC can considerably vary with the production method, giving rise to a wide range of functionalized NFC materials. Moreover, post-production functionalization of NFC is employed during the development of high value-added NFC materials for diverse applications, such as biomedicine, food packaging and electronics [17]. It is expected that the different surface modifications and production methods will influence the physicochemical properties of NFC and, in turn, the interactions with biological systems [10]. This highlights the importance of a thorough characterization of the NFC materials as part of the in vitro hazard assessment. 

**Figure 2:**
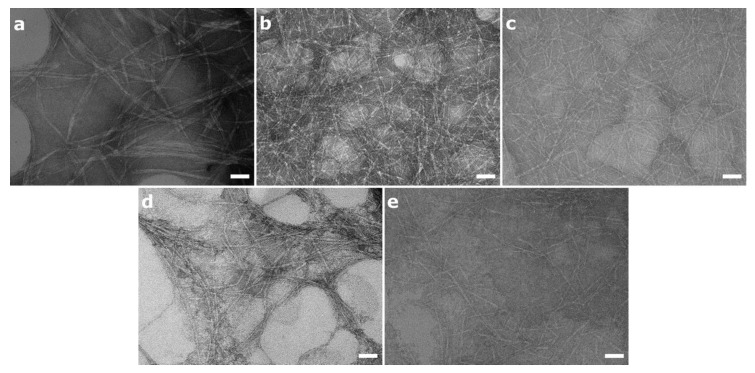
Representative TEM images of NFC aqueous suspensions depicting the morphology of the nanofibers. (**a**) U-NFC, (**b**) A-NFC, (**c**) C-NFC, (**d**) S-NFC and (**e**) P-NFC. Scale bars represent 50 nm.

Table 1 summarizes the physicochemical characteristics of the NFC materials under study, and Figure 2 shows the structural morphology of the different NFCs observed by TEM. 

The surface groups introduced during the production of NFC were quantified, showing similar surface group density between the functionalized materials, with the exception of P-NFC that presented approximately two times higher surface density than the other materials. U-NFC, the non-functionalized material obtained by mild-enzymatic pretreatment, presented a low level of carboxyl groups most probably due to the presence of residual hemicellulose. 

The z-potential values of the NFC materials suspended in 10 mM NaCl, pH 7.5, confirmed the presence of the surface charged groups, with positive values for C-NFC and negative values for the rest of the samples. The differences in surface group density were also reflected in the absolute value of the z-potentials. When the NFCs were suspended in complete cell culture medium, the z-potential values were negative for all materials (around −9 mV). This change in z-potential is explained by the presence of proteins and other biomolecules in the cell culture medium which adsorb to the nanofibers, modifying the material surface and generating a new interface, the so-called protein corona [26]. The characteristics of the protein corona (protein type, amount and conformation), and thus the surface properties of the NFC material when suspended in biological media, will depend on the nanofiber surface chemistry and also on the composition of the media [26]. The effect of media composition on NFC surface characteristics is reflected in the distinct z-potential values of NFC suspended in the different bacteria broths and cell culture medium (Table 1). 

The degrees of crystallinity of A-NFC, C-NFC and S-NFC were similar to the value obtained for U-NFC, being between 52 and 60%; thus, the crystalline structure did not seem to be significantly affected by the functionalization. P-NFC presented the lowest degree of crystallinity of all NFCs, corresponding to 45%, which could indicate that the phosphorylation did affect NFC crystalline domains. 

TEM images of the NFC suspensions showed that the introduction of surface charged groups had a marked effect on fiber morphology (Figure 2). Generally, surface charges are introduced during the production of NFC to facilitate the defibrillation process and are expected to promote electrostatic repulsion between the fibers. As a result, the surface-modified materials are generally characterized by the presence of individual fibrils, while fibers tend to aggregate in the absence of surface modifications. Thus, as expected U-NFC showed 10–30 nm fiber aggregates (Figure 2a), while individual nanofibrils, 4–5 nm in diameter, were observed for A-NFC, C-NFC and P-NFC (Figure 2b,c,e). Slight fibril aggregation was observed in the S-NFC suspension (Figure 2d). This difference in fiber morphology between S-NFC and the other functionalized NFC materials could be related to the slightly lower surface-charged group density of S-NFC compared with the other surface-modified NFC materials (Table 1). 

We have previously shown that fiber morphology is influenced by the presence of proteins, observing fiber agglomeration when functionalized NFC materials were suspended in cell culture media [27].

The Caco-2 cell line was selected to evaluate the in vitro toxicity of the NFC materials toward human intestinal cells. The studies included the evaluation of cell metabolic activity (resazurin assay), cell morphology and cell membrane integrity (live/dead staining) after 24 and 48 h of exposure to the materials. 

**Figure 3:**
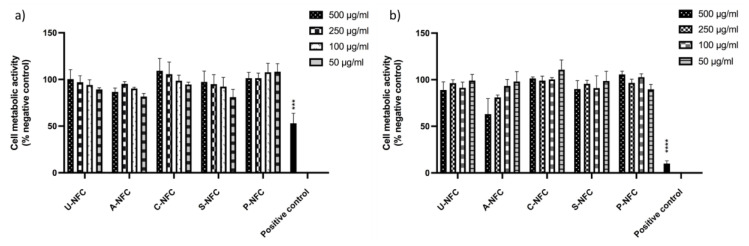
Cell metabolic activity of Caco-2 cells after exposure to the NFC materials. (**a**) 24 h exposure; (**b**) 48 h exposure. Data are expressed as percentage of the negative control (untreated cells) and presented as mean ± SEM of three independent experiments. Significant results as compared with the negative control area marked with asterisks (*** *p* < 0.001, **** *p* < 0.0001).

The resazurin assay results indicated that the metabolic activity of the cells was not significantly affected by the presence of the NFC materials (concentration range 500–50 μg/mL) after 24 and 48 h of exposure (Figure 3). 

It should be noted that, after 48 h exposure to the highest concentration of A-NFC, cells showed a metabolic activity slightly below 70% of the negative control (Figure 3b). However, the difference between the cell metabolic activity of A-NFC exposed cells and non-exposed cells was non-statistically significant.

**Figure 4:**
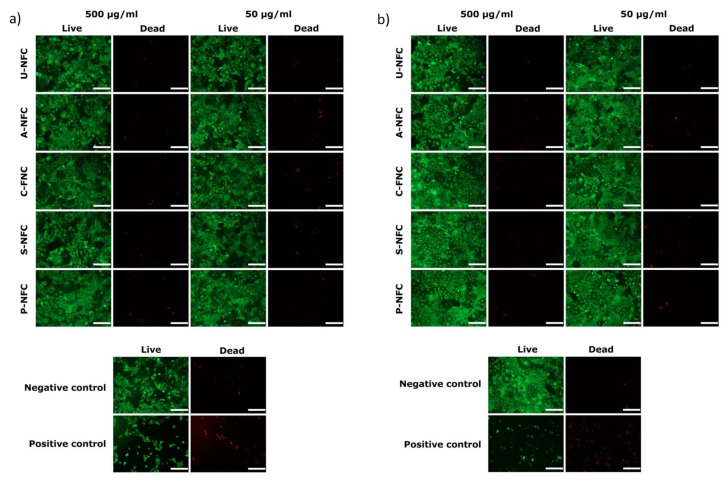
Representative live/dead images of Caco-2 cells after (**a**) 24 h and (**b**) 48 h exposure to the NFC materials. Scale bar represents 100 μm.

The analysis of the microscope images of live/dead stained cells showed that typical cell morphology was maintained in the exposed cells, with a relationship between the number of viable and non-viable cells comparable to the pattern observed in the negative control and significantly different from the positive control images for both 24 h (Figure 4a) and 48 h (Figure 4b) of exposure. Thus, no signs of toxicity in terms of cell membrane damage was observed when cells were exposed to the NFC materials. 

The food industry has begun to exploit the use of nanomaterials to obtain products with extended shelf life, improved quality and higher nutritional value [28]. NFC has long been recognized as a valuable nanomaterial for food-related applications [11]. It has been proposed as stabilizer for food oils and foams, as an additive capable of improving the appearance and sensory properties of food and as a dietary fiber [11,29,30,31]. More recently, scientists have started to investigate the effect of NFC materials in various aspects of the digestion process, such as the intestinal digestion and adsorption of fat [32,33], starch and milk digestion, as well as glucose and mineral adsorption [34,35]. 

Nevertheless, in order to move forward with the development of NFC-containing food products, there is a need to evaluate the toxic effect of NFC in the GIT [11]. Some efforts have been made, in this sense, with Loid et al., who investigated the in vitro and in vivo toxicity of ingested NFC [36], and through the work done by Chen et al., who studied the response of Caco-2 cells exposed to NFC [37]. These toxicological studies focused on unmodified NFC and found no signs of toxicity when Caco-2 cells were exposed to NFC up to 48 h [37], neither when Caco-2 cells were used in an in vitro triculture model and exposed to NFC previously subjected to the conditions of the GIT [36]. The present work expands the type of NFC under study, including different surface modifications to investigate the effect of NFC surface chemistry on the interactions of the nanomaterial with intestinal cells. This becomes relevant, since chemical modifications endow NFC with different physicochemical properties which can influence the interactions with biological systems and thus the nanomaterial´s hazard [38]. For example, it has been shown that the thickness, length and degree of branching of NFC determine the type of interaction with dendritic cells, e.g., internalization vs. frustrated phagocytosis [39]. We have previously found that the inflammatory response of macrophages to NFC materials can be modulated by the introduction of surface-charged groups, with such functionalizations not only affecting the material surface chemistry but also the fiber thickness and aggregation [27]. In the present work, the differences in physicochemical properties of the studied NFCs were not reflected in the Caco-2 response in terms of metabolic activity and cell membrane integrity, with none of the NFCs inducing cytotoxic effects in the intestinal cells. Future studies investigating the effect of the NFC materials on other endpoints, such as oxidative stress and inflammatory response, could help to elucidate other types of cell–NFC interactions.

An important component of the GIT is the normal intestinal flora, with changes in bacteria population being associated with gastrointestinal diseases [13]. Surprisingly, the effect of NFC on the GIT bacteria has not been investigated yet. Thus, in parallel with the intestinal cell studies, the antimicrobial activity of the different NFC materials against normal intestinal flora was investigated. 

**Figure 5:**
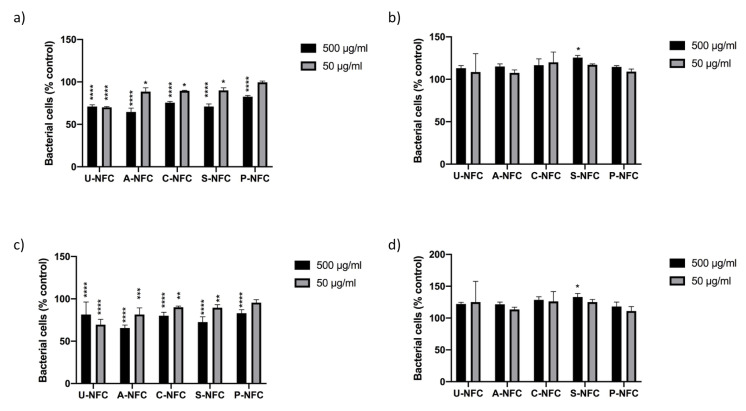
Bacteria survival after exposure to the NFC materials at different time points. (**a**) *E. coli*, 3 h; (**b**) *L. reuteri,* 4 h; (**c**) *E. coli*, 8 h; and (**d**) *L. reuteri*, 8 h. Data are expressed as percentage of the control (untreated bacteria) and presented as mean ± SEM of at least two independent experiments. Significant results as compared with the control area marked with asterisks (* *p* < 0.05, ** *p* < 0.01, *** *p* < 0.001, **** *p* < 0.0001).

The effects of the NFC materials on a Gram-positive (*L. reuteri*) and a Gram-negative (*E. coli*) bacteria were studied by evaluating the bacterial growth curves and by quantifying the CFUs. The bacteria growth curves showed the characteristic growth phases as displayed in Supplementary Materials Figures S1 and S2. Growth curves of *E. coli* exposed to the NFC materials showed certain concentration-dependent growth inhibitory effect (Figure S1), while the growth of *L. reuteri* seemed to be enhanced by the presence of NFC compared with the control (Figure S2). These effects were further investigated by comparing the OD600 values of exposed bacteria to the OD600 values of the controls, at selected time points of the growth curves. The data at both studied time points (4 and 8 h) showed a significant decrease in the number of *E. coli* compared with the control for all NFC materials, with the exception of low concentration P-NFC (Figure 5a,c). On the other hand, the statistical analysis of *L. reuteri* data showed no significant differences between NFC-exposed bacteria and the control, with the exception of high-concentration S-NFC that showed a statistically significant increase in bacteria number compared with the control at both time points (3 and 8 h) (Figure 5b,d). 

Thus, the statistical analysis confirmed a growth inhibitory effect of the NFC samples on *E. coli*, while the growth of *L. reuteri* was not affected by the presence of NFC. 

**Figure 6:**
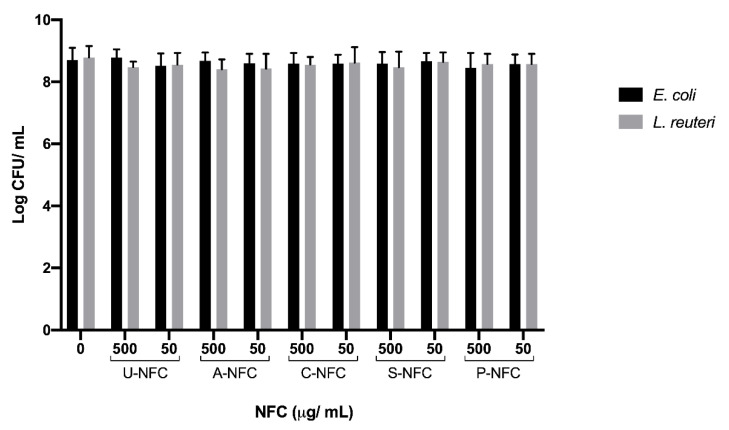
Counts (mean log CFU/mL) for *E. coli* and *L. reuteri* bacteria after exposure to the NFC materials. Data are presented as mean ± SEM of three independent experiments No statistically significant differences were found between NFC-treated and non-treated bacteria.

The effect of NFC on *E. coli* and *L. reuteri* was further examined by the viability assessment done by counting the CFUs of bacteria previously exposed to NFC. Results showed no significant differences between bacteria treated with the NFC materials and the non-treated control (Figure 6). 

This result, together with the observed effect on *E. coli* planktonic growth, may indicate that *E. coli* recovered from the NFC treatment and started to reproduce normally when plated in agar plates and allowed to form colonies. Therefore, the NFC materials under study had a bacteriostatic effect on *E. coli*, while no significant effect on the growth of the Gram-positive bacterium *L. reuteri* was observed. This distinct response between the different types of bacteria may be related to the Gram-character, i.e., the different cell wall structure that could have influenced the bacteria susceptibility to the NFC materials. It is generally stated that Gram-positive bacteria are more sensitive to toxins and antibiotics than Gram-negative bacteria because the uptake of antibiotics into Gram-negative bacteria is slowed down by the presence of the outer membrane [40]. However, the thick and rigid peptidoglycan layer present in Gram-positive bacteria could provide more protection toward certain types of antibacterial agents (e.g., nanomaterials and physical agents) than the thin peptidoglycan layer in Gram-negative bacteria [41,42]. The latest seems to be the case here, where the Gram-negative bacterium *E. coli* was more sensitive to the NFC materials. 

The interactions between surface-modified NFCs and bacteria have mainly been studied with pathogenic bacteria [43,44,45,46,47]. Authors have shown that NFC modified with aldehyde groups and quaternary ammonium groups showed good antibacterial activity against Gram-positive pathogen bacteria [43,46]. Saini et al. found that C-NFC had antimicrobial properties against Gram-positive bacteria, but no antibacterial activity was observed against *E. coli* [43]. On the contrary, here we showed that C-NFC was not able to affect the growth of Gram-positive bacterium, while *E. coli* was more sensitive to the presence of C-NFC. Such differences between the studies could be due to the type of antibacterial test used (contact antibacterial activity vs. inhibition of planktonic growth), the form of C-NFC (film vs. gel suspension) and the degree of substitution of the modified NFC. 

In the work done by Jack et al., carboxylated NFC was subjected to antibacterial tests, showing that the NFC suspension was able to inhibit the growth of the Gram-negative bacterium *P. aeruginosa* when tested at high concentrations (up to 8 mg/mL) [45]. The results of Jack et al. also highlighted the different antibacterial properties of carboxylated NFC when tested as gel suspension, aerogel or film, since the aerogel and film forms of the material did not affect the planktonic growth of the bacterium [45]. The aforementioned differences between different studies highlight the importance of case-by-case study, in terms of NFC material, type of bacteria under study and study design. 

Overall, the present work showed that the differences in NFC surface chemistry were not reflected in the evaluated cell responses, with no indication of cytotoxicity after cell exposure to the NFC materials. A bacteriostatic effect on *E. coli* was observed, independently of the NFC surface chemistry, while the materials did not affect the growth of *L. reuteri*. Further studies need to be done to understand the mechanism behind the distinct response observed with the different types of bacteria. 

## 4. Conclusions

This study presented the first in vitro screening of the interaction of different surface functionalized NFC materials with intestinal cells and microbiota bacteria models. It was shown that neither unmodified NFC nor surface functionalized NFCs impaired the viability of Caco-2 cells. A bacteriostatic effect of the NFC materials toward *E. coli* was found, but no effect on the growth of *L. reuteri* was observed. The findings of this work are expected to contribute to fill in the hazard data gaps regarding NFC oral toxicity and add to the development of safe NFC-based food products.

## Figures and Tables

**Table 1:** Characteristics of the NFC materials under study.

					Z-Potential (mV)		
NFC Sample	Surface Modification	Content Functional Groups (mol/g)	Degree of Crystallinity (%)	NaCl ^b^	MEM ^c^	LB ^d^	MRS ^e^
U-NFC	None	30 ^a^	61	−10.9 ± 2.5	−8.7 ± 1.3	−8.9± 0.7	−6.6± 0.8
A-NFC	Carboxymethylation	570	52	−24.6 ± 2.2	−9.2 ± 1.1	−13.0 ± 0.8	−6.6 ± 0.7
C-NFC	Hydroxypropyl-trimethylammonium substitution	634	61	17.4 ± 2.2	−9.6 ± 0.7	−11.7 ± 0.9	−4.5 ± 0.2
P-NFC	Phosphorylation	1109	45	−31.1 ± 1.2	−9.5 ± 0.4	−16.3 ± 1.6	−14.5 ± 0.6
S-NFC	Sulfoethylation	444	56	−23.8 ± 1.6	−9.8 ± 1.1	−11.8 ± 0 .6	−6.2 ± 0.1

^a^ Residual carboxyl groups; ^b^ determined in 10 mM NaCl at 25 °C, pH 7.5; ^c^ determined in complete MEM cell culture medium at 37 °C, pH 7.8–7.9; ^d^ determined in LB broth at 37 °C, pH 6.8–6.9; ^e^ determined in MRS broth at 37 °C, pH 5.6–5.8.

## Supplementary Materials

The following are available online at https://www.mdpi.com/2079-4991/10/6/1159/s1. Figure S1: Growth curves of *E. coli* exposed to the different nanofibrillated cellulose materials and non-exposed bacteria (control). Figure S2: Growth curves of *L. reuteri* exposed to the different nanofibrillated cellulose materials and non-exposed bacteria (control).
