# Supplementary material for: In Vitro Biological Impact of Nanocellulose Fibers on Human Gut Bacteria and Gastrointestinal Cells
Source: Nanomaterials (Basel). 2020 Jun 12;10(6):1159. doi: 10.3390/nano10061159 (PMC7353236; doi:10.3390/nano10061159)
Supplement: Supplementary file 1 [file nanomaterials-10-01159-s001.pdf]

Supplementary Information

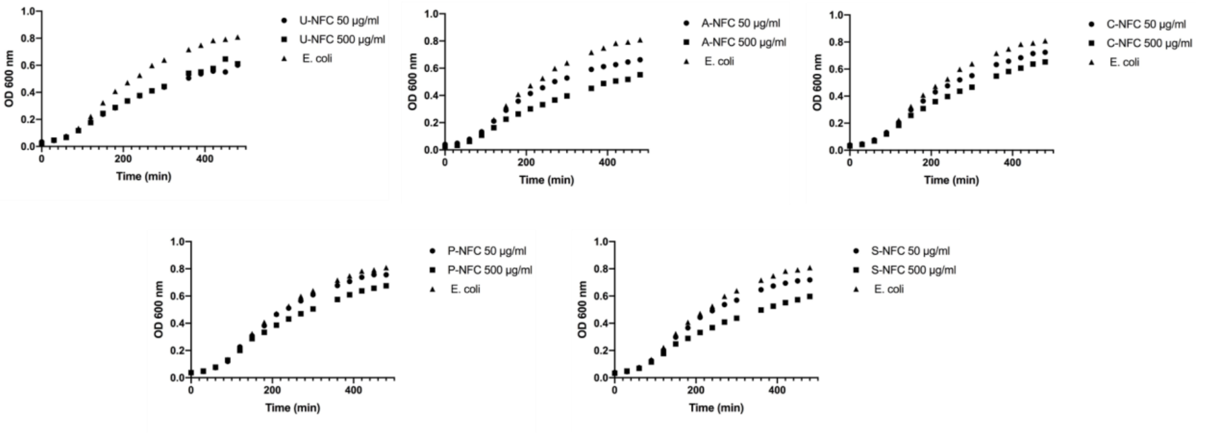

Figure S1. Growth curves of *E.coli* exposed to the different nanofibrillated cellulose materials and non-exposed bacteria.

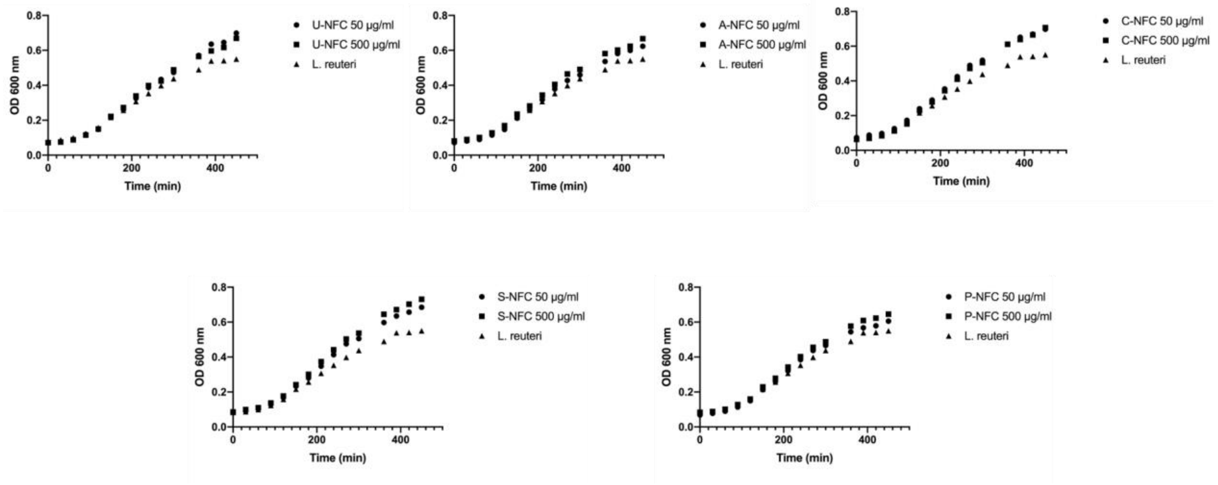

Figure S2. Growth curves of *L.reuteri* exposed to the different nanofibrillated cellulose materials and non-exposed bacteria.
